# The longitudinal course of posttraumatic stress disorder symptoms in war survivors: Insights from cross‐lagged panel network analyses

**DOI:** 10.1002/jts.22795

**Published:** 2022-01-14

**Authors:** Pascal Schlechter, Jens H. Hellmann, Richard J. McNally, Nexhmedin Morina

**Affiliations:** ^1^ Department of Psychiatry University of Cambridge Cambridge UK; ^2^ Institute of Psychology, University of Münster Münster Germany; ^3^ Department of Psychology Harvard University Cambridge Massachusetts USA

## Abstract

Many war survivors suffer from chronic posttraumatic stress disorder (PTSD). Unraveling the complexities of PTSD symptoms over time is crucial for understanding this condition. Going beyond a common pathogenic pathway perspective, we applied the network approach to psychopathology to analyze longitudinal data from war survivors with PTSD in five Balkan countries approximately 8 years after war in the region and a follow‐up assessment 1 year later (*N* = 698). PTSD diagnosis was established using the Mini‐International Neuropsychiatric Interview, and PTSD symptoms were assessed using the Impact of Events Scale–Revised. Undirected cross‐sectional networks for baseline and follow‐up revealed no differences in the overall connectivity between these two networks. The intrusion symptom “I had waves of strong feelings about it” had the strongest expected influence centrality. Directed cross‐lagged panel network models indicated that hyperarousal symptoms predicted other PTSD symptoms from baseline to follow‐up, whereas several avoidance symptoms were predicted by other PTSD symptoms. The findings underscore the importance of emotional reactions and further suggest that hyperarousal symptoms may influence other PTSD symptoms. Future research should investigate causality and associations between between‐person and within‐person networks.

Organized mass violence around the globe can result in debilitating consequences for the mental health of war survivors (Hoppen et al., 2021). Epidemiological research suggests that as many as 227,000,000 adult war survivors may suffer from posttraumatic stress disorder (PTSD; Hoppen et al., 2021). Although the spontaneous remission of PTSD can occur (Kessler et al., 2017), it remains chronic in a substantial proportion of war survivors many years after the war (Hoppen et al., 2021). PTSD has diverse presentations (American Psychiatric Association, 2013), but most researchers have used composite symptom scores, implicitly assuming the covariation of PTSD symptoms reflects an underlying common cause (Karstoft et al., 2013). However, this perspective can obscure complex interactions among the symptoms themselves (McNally et al., 2015).

Accordingly, traumatologists have applied network analytic methods (Borsboom & Cramer, 2013) to study PTSD (for a review, see Birkeland et al., 2020). Instead of assuming that symptoms are interchangeable indicators of an underlying common cause, the network perspective regards a disorder as a causal system emerging from the complex interactions among the disorder's constitute symptomatic elements, which can vary in their importance (e.g., centrality or interconnectedness with other symptoms; Borsboom & Cramer, 2013). Although network and factor analytic methods are mathematically interchangeable, they differ drastically in their ontologies and clinical implications (e.g., Borsboom, 2017; McNally, 2021). If symptoms result from a latent common cause, clinicians should target the common cause as if it were a bacterial infection or malignant tumor. On the other hand, if the disorder is an emergent property that arises from a network of interacting elements, targeting the elements and their interaction is therapeutically advisable. In the context of PTSD, individuals process traumatic experiences and their sequelae in a way that produces a sense of imminent threat (Ehlers & Clark, 2000; Schnyder et al., 2015). This is accompanied by states of hyperarousal and involuntarily recurrent intrusive recollections that foster the avoidance of trauma reminders (Ehlers & Clark, 2000). Although neo‐Kraepelinian construals of psychopathology characterize symptoms as reflective of latent common causes, PTSD theorists have often emphasized direct interactions among symptoms (e.g., Keane et al., 1985), a view that has been formalized in network theory and may encourage advances in treatment. Recent studies have highlighted the importance of treating specific symptoms in PTSD (see Gamble et al., 2021, for a study targeting intrusive symptoms). To inform such approaches, researchers first need to identify the core symptoms that maintain PTSD; they can then test the importance of these symptoms in intervention studies (see Fried, 2020, and Henry et al., 2021, for discussions of theory‐informed network analysis and network‐informed interventions).

Network analytic studies on PTSD have advanced this understanding and identified highly central symptoms of PTSD in war survivors, including recurrent thoughts, negative trauma‐related emotions, and intrusive traumatic memories (Sullivan et al., 2018; see also Birkeland et al., 2020, for a review). Moreover, despite interstudy variability in identified core symptoms (Birkeland et al., 2020), the network structure of different populations of war survivors within studies has not been shown to strongly deviate with regard to overall connectivity according to network comparison tests (Fried et al., 2018; Schlechter et al., 2021).

However, most network analytic studies on PTSD in war survivors have been cross‐sectional, depicting associations among symptoms across many participants at a single point in time (Birkeland et al., 2020). Longitudinal studies have been scarce. One rare exception revealed pre‐ to postcombat changes in network connectivity in male Israeli soldiers (Segal et al., 2020). The authors reported stronger overall connectivity of PTSD symptoms when soldiers were assessed 6 months after combat exposure compared to before combat. Although this study illuminated how the overall strength of node‐to‐node edges changed over two assessment points, it did not visualize the fine‐grained dynamics of an evolving network; that is, the research provided two cross‐sectional snapshots of network structure rather than the temporal dynamics of symptoms (Borsboom & Cramer, 2013). In these networks, psychological reactions to triggers emerged as a central symptom that was associated with intrusion and avoidance symptoms.

It is important to discern how such associations unfold over time. For instance, exposure to reminders of a traumatic experience may initially trigger intrusive reexperiencing symptoms of the event. This, in turn, could foster the avoidance of reminders to reduce distress, which may result in emotional numbness. Elucidating such time‐varying associations represents the core purpose of network analyses. For example, one experience‐sampling network analysis study with 96 participants in Israel provided relevant hints to such patterns (Greene et al., 2020). Specifically, one of the main findings was that arousal strongly predicted other PTSD symptoms and negative emotions while the conflict was occurring.

However, these studies have focused on the acute phase of the conflict and examined circumscribed time ranges. To date, less is known about the symptom constellations in individuals with chronic PTSD several years after a war or conflict. Although world mental health surveys indicate high levels of remission in PTSD symptoms (Kessler et al., 2017), the findings from a recent meta‐analysis suggest that PTSD prevalence in war survivors persists for several years following a war, with a mean time of 7 years between the war and PTSD assessment (see Hoppen et al., 2021). This indicates that many individuals continue to suffer for many years. Therefore, it is critical to identify symptoms of PTSD that may maintain or intensify high levels of suffering (McNally et al., 2015). Intrusive symptoms may have many outgoing associations with other symptoms (i.e., predicting them) in individuals with prolonged PTSD because individuals may feel as if the traumatic event is happening again in the present moment (Brewin et al., 2014). This state could initiate the onset of other symptoms. Indeed, Bryant et al. (2017) reported an increase in the connectivity of reexperiencing symptoms 12 months following trauma exposure (Bryant et al., 2017). In this regard, avoidance may be centrally influenced by other symptoms (i.e., having many incoming associations) in prolonged PTSD because it interferes with adaptively processing trauma‐relevant information and may prevent individuals from dealing with their cognitions regarding the trauma (Ehlers & Clark, 2000).

In the present study, we investigated PTSD symptoms in individuals who were exposed to traumatic events during the Balkan war in the 1990s, which constitutes one of the worst armed conflicts in recent decades. Most of the participants (77.2%) were noncombatant civilians, whereas a minority of the remaining participants were civilians who fought as members of a militia. To enhance understanding of the consequences of this war, we drew on extant longitudinal data regarding war survivors who qualified for a PTSD diagnosis approximately 8 years after the conflict, with a follow‐up assessment 1 year later (Priebe et al., 2013). Other data sets investigating mental health following this war have been limited in their sample composition (e.g., only investigating women; Klarić et al., 2007), sample size (Morina & Ford, 2008), or recruitment method (e.g., target sampling; Başoğlu et al., 2005). The present study addressed these issues via random recruitment of a large number of participants for face‐to‐face interviews along with a 1‐year follow‐up for those with PTSD (Priebe et al., 2013). Given that chronic PTSD trajectories tend to be stable (Solomon et al., 2012), this sample allowed us to investigate a broader period of 1 year compared to only a few months to pinpoint symptoms that maintain PTSD and symptom constellations that favor symptom chronicity. First, we compared the cross‐sectional networks at baseline and follow‐up to identify highly central symptoms and explore changes in their overall connectivity and symptom centrality. Second, we used cross‐lagged panel network (CLPN) models to elucidate time‐varying associations (Rhemtulla et al., in press). CLPN integrates cross‐lagged panel models in the network analysis framework and estimates autoregressive and cross‐lagged pathways of symptoms across time. Given its importance in PTSD theories (Ehlers & Clark, 2000; Schnyder et al., 2015) as well as in an experience sampling study (Greene et al., 2020), we expected that in the CLPN, hyperarousal symptoms would predict other symptoms, whereas avoidance symptoms would be the recipient of activations issuing from other symptoms (Ehlers & Clark, 2000; Greene et al., 2020). Other than testing these hypotheses, our study is exploratory, especially as longitudinal network studies of PTSD are scarce (cf. Schlechter et al., 2021).

## METHOD

### Participants and procedure

The present study builds on the CONNECT project, a multisite European study on the consequences of war and migration on mental health (Bogic et al., 2013; Priebe et al., 2010). Relevant national ethics committees approved the study protocol, including the procedure for securing the written informed consent of all participants (Priebe et al., 2013). The data were collected from mostly civilian individuals who had experienced potentially traumatic events during the war in the Balkan region and had either stayed in the countries of conflict, (i.e., Bosnia–Herzegovina, Croatia, Kosovo, Macedonia, and Serbia) or fled to one of three Western European countries (i.e., Germany, Italy, and the United Kingdom). Participants were recruited in 2006 and 2007. In the countries of former conflict, recruitment followed a multistage probabilistic sampling frame and random walk approach in regions directly exposed to war‐related events. One fifth of these regions within each country were randomly selected. Subsequently, three localities within each of these countries were chosen, resulting in a total of 15 regions and 49 localities. However, data assessment using a random walk sampling recruitment method in Germany, Italy, and the United Kingdom was not feasible because there were no areas with a sufficient density of war survivors who had been born within the former Yugoslavia (Bogic et al., 2013). In the initial study, 4,167 individuals participated; of this initial sample, 3,313 participants were residing in the five Balkan countries and 854 were refugees living in the three Western European countries.

Individuals diagnosed with PTSD received a follow‐up interview 1 year after the baseline interview. Follow‐up data were acquired for 522 of the 655 PTSD‐diagnosed participants residing in one of the Balkan countries and 215 of the 283 PTSD‐diagnosed individuals residing in one of the Western European countries, for a total sample of 737 participants. Due to missing data, information from 698 participants (i.e., 94.7% of the participants assessed at follow‐up) was included in the present analyses. Attrition analyses comparing the sample of individuals who did not participate at follow‐up compared to baseline have been reported elsewhere (Priebe et al., 2013). Concisely, in both samples, more women participated in the follow‐up assessment; among Balkan residential participants, fewer individuals had a secondary‐level education and more reported having vocational or tertiary education. These individuals also reported fewer but more recent experiences of war‐related traumatic events. Given that network analyses require a reasonably large sample size to obtain stable results, we analyzed data from individuals who resided in one of the Balkan countries in combination with data from those who resided in one of the Western European countries. Previous cross‐sectional network analyses on the entire sample have revealed similar global network features between the two groups (Schlechter et al., 2021); hence, we did not expect large differences between the subsamples.

### Measures

All interviews were conducted face to face by 33 trained interviewers (for details, see Bogic et al., 2013, and Priebe et al., 2010).

#### Sociodemographic characteristics

Participants completed a brief sociodemographic questionnaire (see Table 1) at baseline and again during the follow‐up interview 1 year later.

**TABLE 1:** Sociodemographic and trauma‐related characteristics

Characteristic	*n*	%	*M*	*SD*
Sociodemographic data				
Female sex	390	55.8		
Age years			45.6	0.84
Educational attainment (years)			10.33	3.71
Employed at baseline	186	26.6		
Unemployed at baseline	512	73.3		
Living with a partner at baseline	494	70.7		
Trauma‐related data				
Prewar PTEs			0.10	1.25
War‐related PTEs			6.40	3.41
Postwar PTEs			0.09	1.12
Use of health services between baseline and follow‐up				
Use primary care service	610	87.4		
Use mental health care service	268	38.4		

*Note*: *N* = 698. PTE = potentially traumatic event.

#### Potentially traumatic experiences

An adapted 24‐item version of the Life Stressor Checklist–Revised (Wolfe & Kimerling, 1997) was used to assess potentially traumatic experiences that occurred before, during, and after the war. The most frequently endorsed wartime events were lack of food, lack of shelter, shelling, siege, and learning about the violent death of a loved one.

#### PTSD diagnosis

To assess PTSD and other diagnoses, applicable translated versions of the Mini‐International Neuropsychiatric Interview (M.I.N.I.; Sheehan et al., 1998) were employed as a structured diagnostic interview. The MINI is based on psychiatric disorder criteria outlined in the *Diagnostic and Statistical Manual of Mental Disorders* (fourth ed.; *DSM‐IV*) and *International Classification of Diseases* (10th rev.; *ICD‐10*) and has demonstrated good reliability and validity in comparison to the Structured Clinical Interview for *DSM‐III‐R* and Composite International Diagnostic Interview (Lecrubier et al., 1997; Sheehan et al., 1997). Specifically, the M.I.N.I. PTSD module has demonstrated high interrater reliability (κ = .95) and acceptable test–retest reliability (κ = .73). Furthermore, the PTSD module exhibits good sensitivity (.85), specificity (.96), positive predictive value (.82), and negative predictive value (.97; Sheehan et al., 1997). For the data used in this investigation, the mean agreement between the interviewers was 92% (Priebe et al., 2013).

#### Trauma‐related symptoms

Posttraumatic stress symptom levels were measured using the 22‐item Impact of Events Scale–Revised (IES‐R; Weiss, 2004). Respondents were asked to rate symptoms on a 5‐point Likert‐type scale ranging from 0 (*not at all*) to 4 (*extremely*). The IES‐R has demonstrated good internal consistency and high test–retest reliability (Weiss, 2004) and has been translated and validated in the countries comprising the former Yugoslavia (Franciskovic et al., 2008; Ljubotina & Muslic, 2003; Morina, 2003). In the current project, participants first identified at least one war‐related event they considered to be the most disturbing they had experienced and were then asked to rate each IES‐R item with respect to this event. Internal consistency was excellent at both baseline, ω_t_ = .95, Cronbach's α = .94, and follow‐up, ω = .97, Cronbach's α = .96.

### Data analysis

We used R (Version 4.0.3; R Core Team, 2020) to perform all analyses. First, we computed a separate Gaussian graphical model (GGM) for the cross‐sectional networks at baseline and follow‐up. A GGM comprises nodes (i.e., symptoms) connected by edges that signify the partial correlation between pairs of nodes, adjusted for the influence of all other nodes in the network (Borsboom & Cramer, 2013). Second, we ran a CLPN to unravel the connections between the baseline and follow‐up assessments over time (Rhemtulla et al., in press). A CLPN depicts how nodes (i.e., symptoms) at one point predict nodes at a second time point. The weights of these directed edges signify regression estimates. All networks were visualized with the R package *qgraph* (Epskamp et al., 2012). Data were expected to be missing at random (MAR). Given that CLPN networks are incompatible with full‐information maximum likelihood estimation, we based all analyses on complete cases having no missing data at the item level, a sample size of 698. We reasoned that the scarcity of missing values (i.e., ∼5%) would have little effect on the findings. Nevertheless, in a sensitivity analysis, we also conducted the cross‐sectional and CLPN network analyses with a single imputed data set. Using one imputed dataset is recommended for network analysis when missing data are of concern (Rhemtulla et al., in press). To this end, we imputed data by using predictive mean matching with the *MICE* package in R (van Buuren et al., 2015). All results matched those of the full dataset, and conclusions remained unaffected (see Supplementary Figures S1–S5 for this sensitivity analysis). For all networks, we included all IES‐R items.

For the two cross‐sectional networks, we applied the least absolute shrinkage and selection operator (LASSO) to avoid estimating spurious connections (Epskamp et al., 2012). The LASSO includes a regularization term that shrinks small edges to 0 so that only the most robust associations between nodes appear in the networks. For visual comparisons, we used average layouts for both the baseline and follow‐up networks. In these networks, nodes represent symptoms, and edges are estimates of regularized partial correlations between the symptoms. To compare the overall connectivity of these two networks, we used the network comparison test in the *NetworkComparisonTest* package in R (van Borkulo et al., 2016). We computed the expected influence centrality for each symptom, which represents a node's interconnectedness with other nodes (i.e., the sum of the edge weights, both negative and positive, connected to a node; Robinaugh et al., 2016).

Next, we computed a directed CLPN from baseline to the follow‐up assessment (i.e., baseline →follow‐up) by using the *glmnet* package (Friedman et al., 2010). The CLPN permits researchers to model the directed networks for two time points, whereas other established network methods for panel data require at least three time points (Epskamp, 2020). We first calculated regression models to estimate the autoregressive and cross‐lagged coefficients. In autoregressive pathways, a symptom at baseline predicts itself at follow‐up after adjusting for all other symptoms at the first time point. In the cross‐lagged pathways, a symptom at baseline predicts a different symptom at follow‐up after adjusting for all other symptoms at the first time point. As in the cross‐sectional networks, we applied a LASSO penalized maximum likelihood procedure with a 10‐fold cross‐validation tuning parameter that shrunk small regression coefficients to 0. Again, nodes represent symptoms, and arrows represent the estimates of cross‐lagged effects. The color of the arrows in Figure 2 represents the directionality of the effect, with blue arrows indicating positive effects and red arrows negative effects. Line thickness signifies the strength of association. The autoregressive paths were the strongest paths in the network and, therefore visually, suppressed the cross‐lagged paths, which is of particular interest for the present analyses. Thus, we set the autoregressive paths to 0 to highlight the cross‐lagged effects most relevant to our study aims (Rhemtulla et al., in press).

For the directed CLPNs, we calculated the following centrality indices: cross‐lagged “in” expected influence (IEI) and “out” expected influence (OEI). The IEI quantifies the degree to which each symptom is predicted by other symptoms in the network (i.e., the sum of the values of incoming edges associated with a symptom), whereas the OEI describes the degree to which each symptom predicts other symptoms in the network (i.e., the values of outgoing edges associated with a symptom).

**FIGURE 1:**
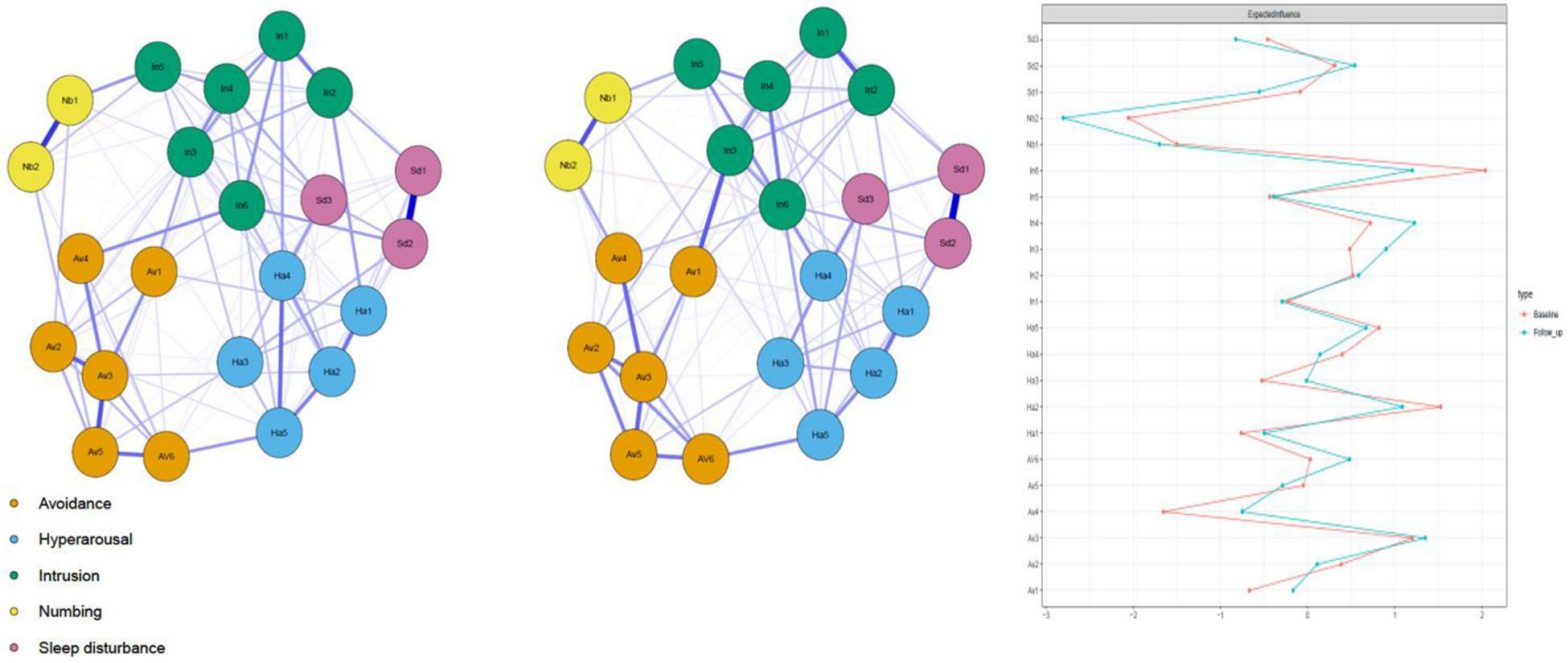
Cross‐sectional networks for baseline (left) and the follow‐up (middle), and expected influence centrality (right), using z values. *Note*. In1 = Any reminder brought back feelings about it; In2 = Other things kept making me think about it; In3 = I thought about it even when I didn't mean to; In4 = Pictures about it popped into my mind; In5 = I found myself acting like I was back at that time; In6 = I had waves of strong feelings about it; Av1 = Avoided letting myself get upset when I thought about; Av2 = I stayed away from reminders of it; Av3 = I tried not to think about it; Av4 = Lot of feelings about it; but didn't deal with them; Av5 = I tried to remove it from my memory; Av6 = I tried not to talk about it; Ha1 = I felt irritable and angry; Ha2 = I was jumpy and easily startled; Ha3 = I had trouble concentrating; Ha4 = Reminders of it caused me to have physical reactions; Ha5 = I felt watchful and on guard; Nb1 = I felt as if it hadn't happened or it wasn't real; Nb2 = My feelings about it were kind of numb; Sd1 = I had trouble staying asleep; Sd2 = I had trouble falling asleep; Sd3 = I had dreams about it

**FIGURE 2:**
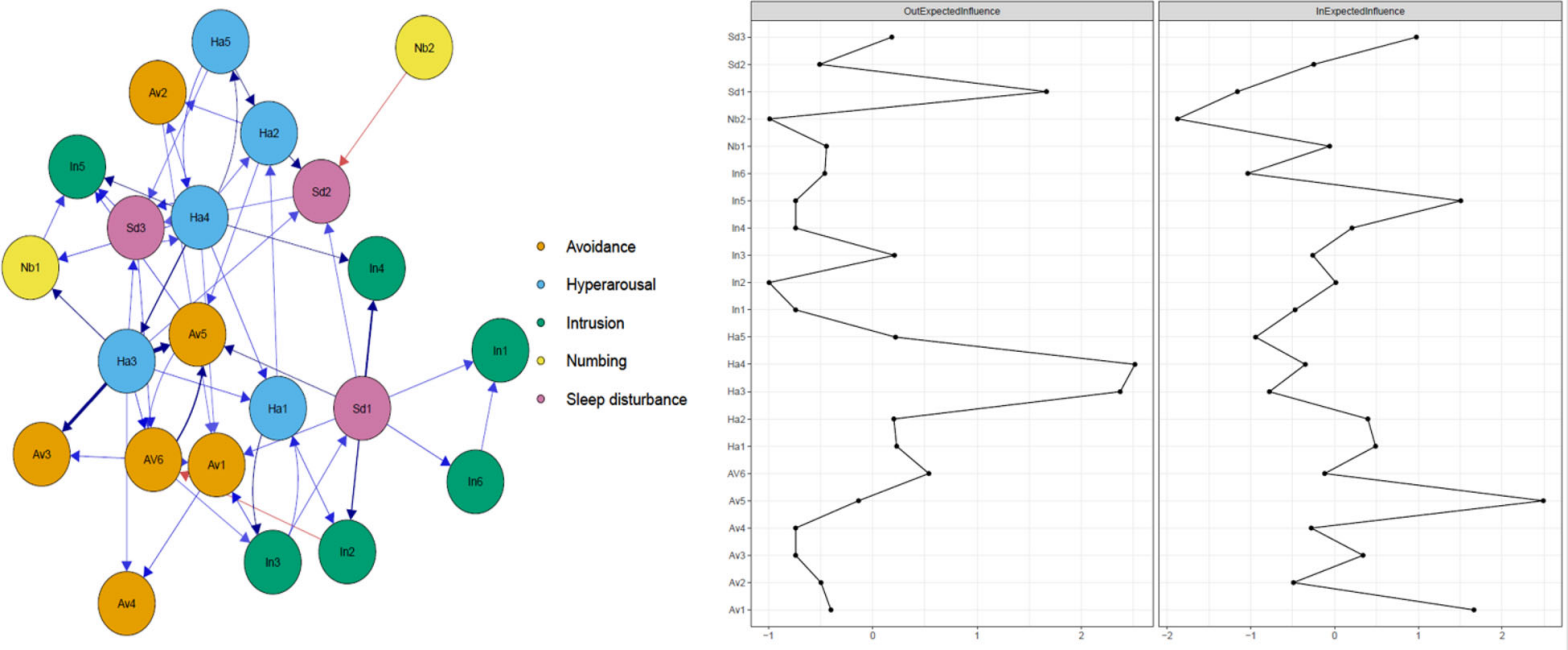
Cross‐lagged panel network (left) and respective centrality estimates (right), using z values. Note. Higher values indicate more centrality. For visualization, a beta threshold of .05 for the regression weights was chosen. In1 = Any reminder brought back feelings about it; In2 = Other things kept making me think about it; In3 = I thought about it even when I didn't mean to; In4 = Pictures about it popped into my mind; In5 = I found myself acting like I was back at that time; In6 = I had waves of strong feelings about it; Av1 = Avoided letting myself get upset when I thought about; Av2 = I stayed away from reminders of it; Av3 = I tried not to think about it; Av4 = Lot of feelings about it; but didn't deal with them; Av5 = I tried to remove it from my memory; Av6 = I tried not to talk about it; Ha1 = I felt irritable and angry; Ha2 = I was jumpy and easily startled; Ha3 = I had trouble concentrating; Ha4 = Reminders of it caused me to have physical reactions; Ha5 = I felt watchful and on guard; Nb1 = I felt as if it hadn't happened or it wasn't real; Nb2 = My feelings about it were kind of numb; Sd1 = I had trouble staying asleep; Sd2 = I had trouble falling asleep; Sd3 = I had dreams about it

In all networks, to enhance the visual interpretability of Figures 1 and 2, we colored nodes according to the previously identified factorial solution of the IES‐R (i.e., intrusion, avoidance, hyperarousal, numbing, and sleep disturbance; Morina et al., 2010). To estimate the accuracy of edge weights, we placed 95% confidence intervals around each edge weight with nonparametric bootstrapping (1,000 iterations). In addition, we calculated the correlation stability coefficient (CS‐coefficient), which ranges from 0 to 1, whereby strong stability is indicated when values of .50 or higher. To this end, we used the *bootnet* package (Epskamp et al., 2018). Further, we used the edge weight difference test and centrality difference test to examine whether edges and centrality indices differed significantly from each other (Epskamp et al., 2018).

## RESULTS

The mean values and standard deviations for all IES‐R items, as assessed at baseline and follow‐up, are presented in Table 2 along with the skewness and kurtosis for each symptom. Mean symptom levels decreased from baseline to follow‐up, as evinced by a significant decrement in total scores, *t*(697) = −12.92, *p* ˂ .001, Hedges’ *g *= −0.492. The skewness and kurtosis for all items were within an acceptable range and did, thus, not indicate any deviation from normality.

**TABLE 2:** Descriptive statistics at baseline and follow‐up

	T1				T2			
Variable	*M*	*SD*	Skew	Kurtosis	*M*	*SD*	Skew	Kurtosis
Any reminder brought back feelings about it	2.89	1.06	−0.92	0.43	2.36	1.20	−0.38	−0.75
I had trouble staying asleep	2.59	1.25	−0.64	−.055	2.17	1.36	−0.30	−1.12
Other things kept making me think about it	2.60	1.13	−0.65	−0.21	2.16	1.25	−0.26	−0.91
I felt irritable and angry	2.30	1.28	−0.36	−0.92	1.90	1.32	−0.05	−1.12
Avoided letting myself get upset when I thought about	2.58	1.11	−0.62	−0.15	2.19	1.24	−0.35	−0.86
I thought about it even when I didn't mean to	2.58	1.13	−0.65	−0.19	2.15	1.26	−0.35	−0.95
I felt as if it hadn't happened or it wasn't real	1.52	1.23	0.33	−1.33	1.24	1.33	0.59	−1.03
I stayed away from reminders of it	2.52	1.23	−0.61	−0.51	2.11	1.33	−0.26	−1.12
Pictures about it popped into my mind	2.80	1.15	−0.88	0.04	2.24	1.28	−0.37	−0.92
I was jumpy and easily startled	2.65	1.24	−0.75	−0.38	2.11	1.32	−0.26	−1.04
I tried not to think about it	2.68	1.18	−0.79	−0.10	2.17	1.30	−0.28	−1.00
Lot of feelings about it, but didn't deal with them	2.31	1.26	−0.37	−0.82	1.82	1.32	−0.01	−1.17
My feelings about it were kind of numb	1.37	1.36	0.48	−1.11	1.19	1.28	0.65	−0.87
I found myself acting like I was back at that time	2.02	1.39	−0.15	−1.24	1.61	1.42	0.27	−1.32
I had trouble falling asleep	2.73	1.25	−0.77	−0.38	2.29	1.37	−0.37	−1.09
I had waves of strong feelings about it	2.92	1.14	−0.84	−0.03	2.15	1.33	−0.30	−1.07
I tried to remove it from my memory	2.55	1.30	−0.69	−0.57	2.02	1.40	−0.12	−1.26
I had trouble concentrating	2.44	1.25	−0.46	−0.79	1.88	1.35	−0.02	−1.21
Reminders of it caused me to have physical reactions	2.56	1.31	−0.60	−0.71	2.01	1.43	−0.09	−1.34
I had dreams about it	2.30	1.43	−0.38	−1.17	1.73	1.45	0.15	−1.35
I felt watchful and on guard	2.51	1.25	−0.57	−0.63	1.94	1.38	−0.07	−1.25
I tried not to talk about it	2.54	1.28	−0.62	−0.63	2.09	1.41	−0.14	−1.27
IES‐R total score	2.45	0.81	−0.58	0.32	1.98	1.00	−0.28	−0.77

*Note*: *N* = 698. IES‐R = Impact of Event Scale–Revised.

### Cross‐sectional networks

Accuracy plots showed small‐to‐moderate confidence intervals, suggesting good accuracy for the baseline and follow‐up networks (see Supplementary Figures S6 and S7). Likewise, the case‐drop bootstrapping results indicated strong stability of the expected influence centrality measures (Supplementary Figures S8 and S9). Overall, the two networks, which are depicted in Figure 1 with their expected influence centrality plots, showed many consistencies. There were no significant differences in overall connectivity between the networks according to the network comparison test, *S *= 0.448, *p *= .070. In the baseline network, the number of nonzero edges was 131 out of 231, and in the follow‐up network, the number of nonzero edges was 134 out of 231.

The edge lists of the two networks were strongly correlated, *r* = .82. The entire edge list for both networks is given in the Supplementary Materials (i.e., edgst1 and edgst2). In the baseline network, the strongest edges were between the symptoms *I had trouble staying asleep* and *I had trouble falling asleep, I felt as if it hadn't happened or it wasn't real* and *my feelings about it were kind of numb*, and *I tried not to think about it* and *I tried to remove it from my memory*. The first two edges were also the strongest in the follow‐up network, whereas the third strongest edge was between the items *I thought about it even when I didn't mean to* and *I avoided letting myself get upset when I thought about it*. These edges were significantly stronger than many other edges in the network, as indicated by the edge weight difference plots for both networks (see Supplementary Figures S10 and S11).

The correlation between expected influence centralities was also high, *r* = .91. In both networks, the intrusion symptom *I had waves of strong feelings about it* emerged as the strongest symptom. Also, *I was jumpy and easily startled* and *I tried not to think about it* both demonstrated relatively high expected influence centrality. The numbing symptoms *I felt as if it hadn't happened or it wasn't real* and *My feelings about it were kind of numb* demonstrated relatively low values, which were strongly interrelated but somewhat isolated in both networks. Overall, these symptoms had significantly higher and lower expected influence centrality, respectively, than many other symptoms within the networks, evinced by the expected influence centrality difference plots (see Supplementary Figures S12 and S13).

### Cross‐lagged panel network

The accuracy plots indicated high accuracy of the CLPN (see Supplementary Figure S14). In addition, the case‐drop bootstrapping results suggested strong stability for the IEI and OEI (see Supplementary Figure S15).

Figure 2 shows the CLPN for baseline symptoms predicting follow‐up symptoms along with the centrality plots for the IEI and OEI. All edge weights are given in the Supplementary Materials (i.e., edgstCLPN). The symptom constellation *I had trouble concentrating* → *I tried to remove it from my memory* displayed the strongest cross‐lagged connection, followed by the connections between *I had trouble concentrating* → *I tried not to think about it* and *I had trouble staying asleep* → *Pictures about it popped into my mind*. The fourth and fifth strongest cross‐lagged edges were found between *I tried not to talk about it → I tried to remove it from my memory* and *Reminders of it caused me to have physical reactions → I had trouble concentrating*, respectively. The edge weights difference tests indicated that these edges were significantly stronger than most other edges.

Descriptively, the symptoms *Reminders of it caused me to have physical reactions, I had trouble concentrating*, and *I had trouble staying asleep* had the highest OEI, whereas the symptoms *Avoided letting myself get upset when I thought about it, I tried to remove it from my memory, I found myself acting like I was back at that time* and *I had dreams about it* had the highest IEI. These symptoms displayed significantly higher OEI and IEI, respectively, compared to other symptoms in the CLPN (see Supplementary Figures S16 and S17 for difference plots).

## DISCUSSION

For the present study, we compared the overall connectivity of two cross‐sectional PTSD networks in a sample of survivors of the Balkan war 8 years postconflict and at a follow‐up assessment 1 year later. Additionally, we computed one CLPN for the two consecutive assessments to disentangle ingoing and outgoing connections between PTSD symptoms. The two cross‐sectional undirected networks displayed similar structures, as reflected by the many consistent edges, high correlations of edge weights and expected influence centrality, and no significant differences in overall connectivity. We found lower mean total symptom scores at follow‐up compared to baseline (see Priebe et al., 2013). Overall, the observed decrease in symptom severity is in line with findings from naturalistic prospective studies, which suggest that symptoms often decline over time (Morina et al., 2014). However, significant symptom improvement would ideally be accompanied by lower overall connectivity in the follow‐up network to reduce the likelihood of spontaneous relapse. This is because the activation of a core symptom may, in theory, activate large parts of the entire network (Borsboom & Cramer, 2013). Because a reduction in total symptom score does not imply reduced connectivity, connectivity would ideally decrease in parallel with total scores. That is, when connectivity fails to diminish, individuals may remain vulnerable to the reemergence of symptoms. If one symptom were to reactivate, others may follow, producing a cascade of symptom activation and, hence, a relapse of PTSD. Consistent with this claim, van Borkulo et al. (2015) found that more versus less dense networks at baseline predicted persistent versus remitted depression at follow‐up. Accordingly, the similarity in the present PTSD networks may be attributable to participants still qualifying for a PTSD diagnosis 8 years after the war. In fact, approximately 62% of the sample had not used mental health services between baseline and follow‐up. Effective treatment might have attenuated connectivity by counteracting avoidance behavior that might otherwise follow from reexperiencing symptoms. However, empirical evidence regarding connectivity and symptom reduction for different disorders is mixed, especially in within‐participant study designs (Beard et al., 2016; Fried et al., 2016). In one treatment study, the overall connectivity in a comorbid eating disorder, depression, and anxiety network did not change over time, whereas symptom severity decreased; however, less improvement was associated with more densely connected networks at treatment admission (Smith et al., 2019). It may, therefore, be that network connectivity is a valid between‐group discriminator for relapse likelihood at longer follow‐up periods instead of being generally associated with symptom reduction in within‐study designs.

In both networks, the symptom *I had waves of strong feelings about it* emerged as the symptom with the highest expected influence centrality. This intrusion symptom has emerged as central in other PTSD studies (for a review, see Birkeland et al., 2020). This is in line with cognitive models of PTSD that depict intrusions as a potential driver of other symptom clusters (Brewin, 2014; Ehlers & Clark, 2000). Furthermore, avoidance symptoms played a central role in both networks, as reflected in strong edge weights and high centrality of the symptom *I tried not to think about* *it*, consistent with the notion that avoidance is a central feature in the maintenance of PTSD (Ehlers & Clark, 2000). In addition, the symptom *I was jumpy and easily startled* was central. These identified symptoms are often referred to as the core of PTSD symptom presentation (Ehlers & Clark, 2000; Schnyder et al., 2015). As with former network analyses, these findings are in accord with clinically informed theories of PTSD (e.g., Bryant et al., 2017) and further corroborated by the longitudinal networks.

The CLPN revealed that hyperarousal symptoms often predicted other symptoms, consistent with the symptoms *Reminders of it caused me to have physical reactions* and *I had trouble concentrating* having the highest OEI. This is in accord with experience sampling network analyses that have shown that arousal strongly predicts other PTSD symptoms and negative emotions in a war‐affected population (Greene et al., 2020) as well as with treatment studies in which physiological reactivity was shown to predict distress reactivity and flashbacks (Hoffart et al., 2019). Hyperarousal may foster the dysfunctional processing of cues related to a traumatic experience, thereby prompting other symptoms. In fact, these symptoms were highly predictive of the avoidance symptoms *I tried to remove it from my memory* and *I tried not to think about it*, consistent with *Avoided letting myself get upset when I thought about it* and *I tried to remove it from my memory* having high IEI. Together with the high centrality in the cross‐sectional networks, these results suggest that avoidance shows a central downstream effect potentially initiated by hyperarousal. Indeed, arousal may signify a state of hypersensitivity to threat, which, in turn, may increase intrusive symptoms, such as the in‐strength connections to *I found myself acting like I was back at that time* (Greene et al., 2020). These sensations alongside poor contextual memory integration may lead to feelings of immediate threat, followed by avoidance responses to counteract overwhelming trauma‐related sensations (Brewin et al., 2014; Ehlers & Clark, 2000).

Although such pathways appear plausible, *Reminders of it caused me to have physical reactions* were nosologically conceptualized as a measure of *DSM‐IV* reexperiencing and *DSM‐5* intrusion (American Psychiatric Association, 2013). In *DSM‐5* Cluster B, this symptom is regarded as the first indication of recurrent, intrusive, and distressing recollections of the event. An alternative framework in which this symptom is seen as intrusiveness rather than arousal would be consistent with the conceptualization that intrusiveness leads to distress and subsequent avoidance (Ehlers & Clark, 2000).

Further, the symptom *I had dreams about it* had many incoming connections that also represented a central downstream effect of such cascades, whereas *I had trouble staying asleep* had many outgoing connections. This could represent a dysfunctional cycle because sleep disturbance had outgoing and incoming symptom connections and these symptoms may, therefore, mutually have reinforced each other, thereby intensifying sleep problems. These findings align with findings showing that disturbed sleep can be bidirectionally related to different aspects of PTSD (Babson & Feldner, 2010) and that sleep problems are transdiagnostically related to mental distress, making sleep‐related symptoms a pertinent intervention target (Harvey & Buysse, 2017).

Despite the temptation to translate the current findings into clinical practice, researchers must first test whether a reduction in any core symptom predicts lower network activity (see Fried et al., 2018). Still, the present results may inform future intervention studies and theory building for individuals with chronic PTSD. Intervention studies could target hyperarousal or intrusions to examine whether this reduces their impact on other PTSD symptoms. Theoretically, individuals with PTSD may then be less prone to avoid threat‐related stimuli (Ehlers & Clark, 2000). Alternatively, focusing on avoidance directly could have the effect that the network is disrupted because hyperarousal symptoms no longer lead to avoidance and, thus, lose their outgoing centrality. For instance, recent, brief interventions targeting intrusive trauma memories (Gamble et al., 2021) might diminish overall network connectivity.

The current findings should be interpreted in light of the study's limitations. The results are based on only two assessment points, and future research should preferably include more. This is especially relevant because the current study was conducted among individuals with PTSD who had experienced war‐related events 8 years earlier. Although such symptom persistence enables investigators to identify problematic associations between symptoms that maintain PTSD, future studies should incorporate multiple assessment points that occur both before and after the disorder becomes chronic. This may aid in the identification of treatment targets to prevent disorder chronicity upon symptom resolution. Furthermore, the findings need to be replicated in PTSD populations other than war survivors. Altogether, it is important to disentangle different sources of within‐ and between‐person variance, which cannot be fully accomplished with the undirected networks or the CLPN (Rhemtulla et al., in press). Therefore, intensive, longitudinal sampling of symptoms could provide the most informative insights regarding targets for therapeutic intervention (e.g., Greene et al., 2018). Such analyses are also critical because of the likely stable interindividual differences in certain nodes. For instance, hyperarousal symptoms may represent stable nodes. Although symptom assessments at more than two time points would have been beneficial to elucidate more granulated insights (e.g., by estimating contemporaneous networks and disentangling different variance sources; Epskamp, 2020), the present sample constituted the most pertinent sample for the investigations of mental health in the aftermath of the Balkan war.

The results are only unbiased when data are MAR (Rhemtulla et al., in press). Although analyses based on full cases and single imputation converged, we cannot rule out the possibility that unmeasured variables have influenced missingness. Additionally, network centrality indices should be interpreted carefully in the context of psychopathology (Bringmann et al., 2019; Hallquist et al., 2019). Such indices should be understood in the light of the underlying theory and assumptions (e.g., the absence of latent variables in the network model). Simulations indicate that centrality indices can be limited in their meaning when latent confounding is present (Hallquist et al., 2019).

Given its debilitating mental health consequences, understanding PTSD symptoms in war survivors and beyond is a crucial goal for research and clinical practice. Despite mean decreases in symptom levels, global network connectivity remained similar at both assessment points, which may have relevant implications for symptom relapse. The present analyses underline the importance of intrusion symptoms in the undirected networks. Moreover, the CLPN suggests the central role of hyperarousal symptoms in influencing other symptoms, whereas avoidance symptoms seem to be a central end product to deal with severe consequences of trauma exposure.

## OPEN PRACTICES STATEMENT

Neither of the studies reported in this article was formally preregistered. Neither the data nor the materials have been made available on a permanent third‐party archive; requests for the data or materials can be sent via email to the lead author at ps798@medschl.cam.ac.uk

## Supporting information



Supporting Material

Supporting Material

Supporting Material

Supporting Material
