# Supplementary material for: The longitudinal course of posttraumatic stress disorder symptoms in war survivors: Insights from cross‐lagged panel network analyses
Source: J Trauma Stress. 2022 Jan 14;35(3):879–90. doi: 10.1002/jts.22795 (PMC9303894; doi:10.1002/jts.22795)
Supplement: Supplementary file 4 — Supporting Material [file JTS-35-879-s003.docx]

**Supplemental Material 1**


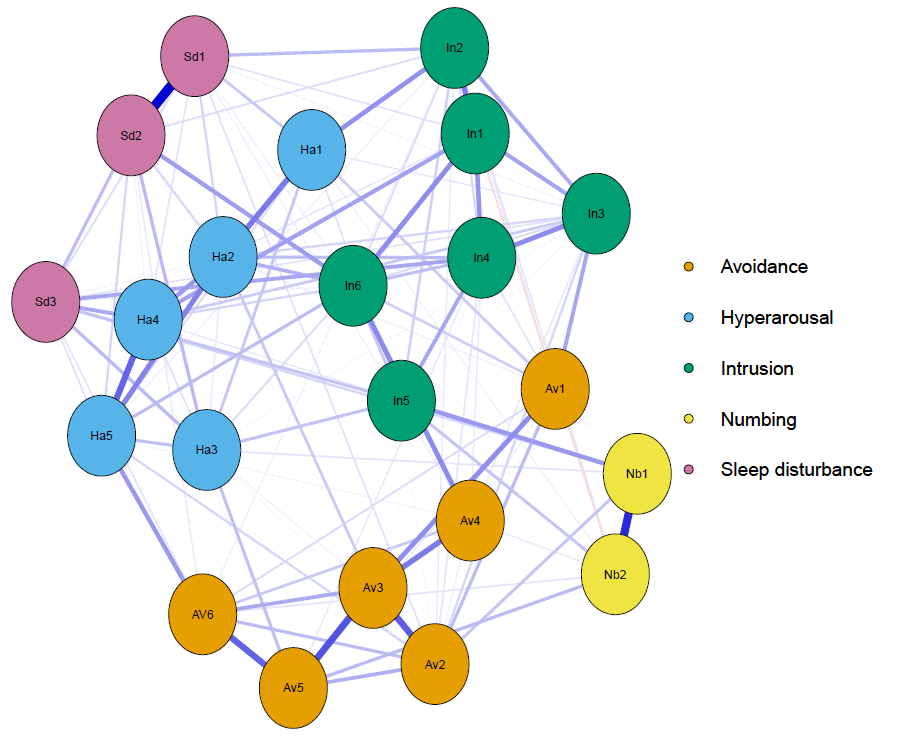


**Supplementary Figure S1**. The cross-sectional network for baseline with a single imputed data set (*N* = 737). In1 = Any reminder brought back feelings about it, In2 = Other things kept making me think about it, In3 = I thought about it even when I didn’t mean to, In4 = Pictures about it popped into my mind, In5 = I found myself acting like I was back at that time, In6= I had waves of strong feelings about it; Av1 = Avoided letting myself get upset when I thought about, Av2 = I stayed away from reminders of it, Av3 = I tried not to think about it, Av4 = Lot of feelings about it, but didn’t deal with them, Av5 = I tried to remove it from my memory, Av6 = I tried not to talk about it; Ha1 = I felt irritable and angry, Ha2 = I was jumpy and easily startled, Ha3 = I had trouble concentrating, Ha4 = Reminders of it caused me to have physical reactions, Ha5 = I felt watchful and on guard; Nb1 = I felt as if it hadn’t happened or it wasn’t real, Nb2 = My feelings about it were kind of numb; Sd1 = I had trouble staying asleep, Sd2 = I had trouble falling asleep, Sd3 = I had dreams about it


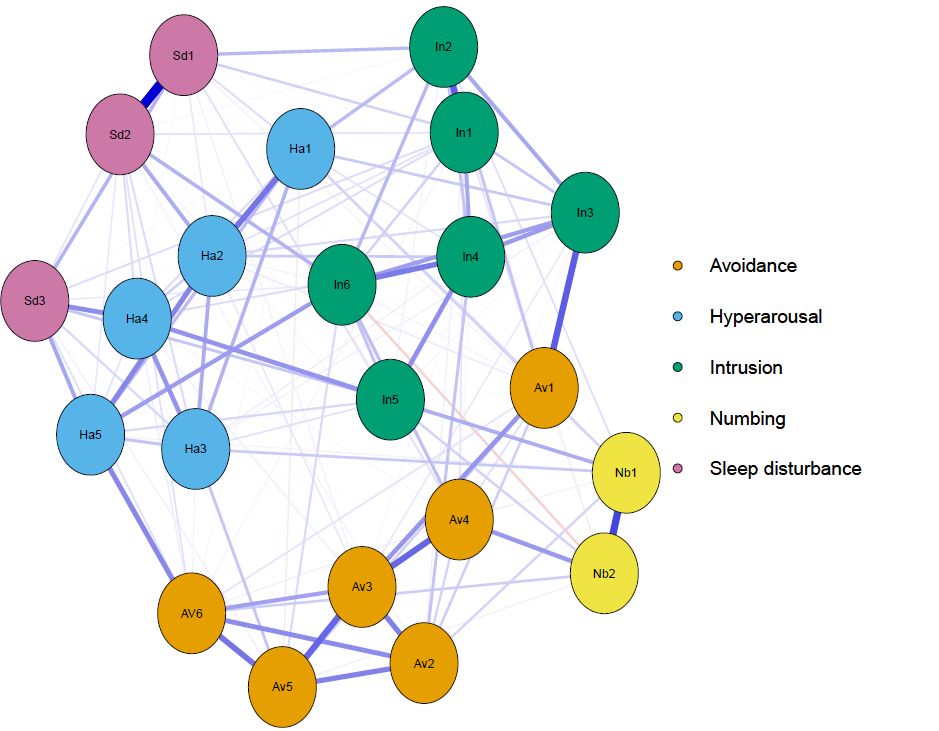


**Supplementary Figure S2**. The cross-sectional network for the follow-up with a single imputed data set (*N* = 737). In1 = Any reminder brought back feelings about it, In2 = Other things kept making me think about it, In3 = I thought about it even when I didn’t mean to, In4 = Pictures about it popped into my mind, In5 = I found myself acting like I was back at that time, In6= I had waves of strong feelings about it; Av1 = Avoided letting myself get upset when I thought about, Av2 = I stayed away from reminders of it, Av3 = I tried not to think about it, Av4 = Lot of feelings about it, but didn’t deal with them, Av5 = I tried to remove it from my memory, Av6 = I tried not to talk about it; Ha1 = I felt irritable and angry, Ha2 = I was jumpy and easily startled, Ha3 = I had trouble concentrating, Ha4 = Reminders of it caused me to have physical reactions, Ha5 = I felt watchful and on guard; Nb1 = I felt as if it hadn’t happened or it wasn’t real, Nb2 = My feelings about it were kind of numb; Sd1 = I had trouble staying asleep, Sd2 = I had trouble falling asleep, Sd3 = I had dreams about it


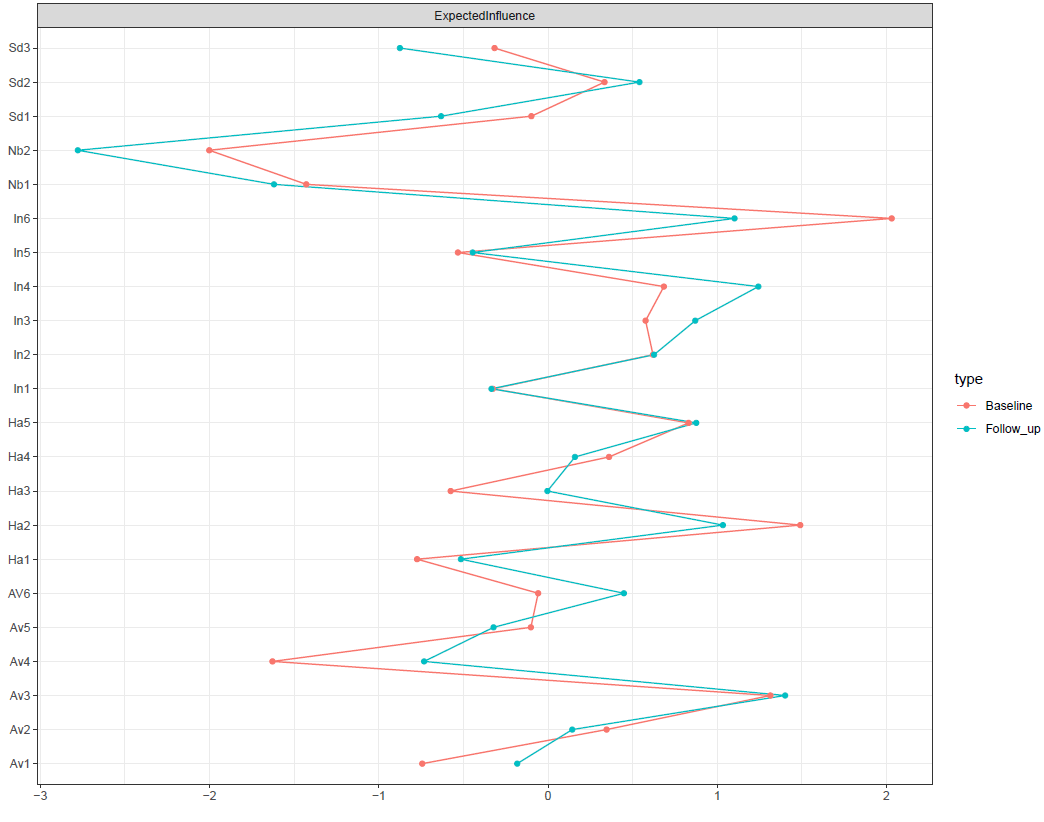


**Supplementary Figure S3***.* The expected influence centrality (right) using z-values with a single imputed data set (*N* = 737). In1 = Any reminder brought back feelings about it, In2 = Other things kept making me think about it, In3 = I thought about it even when I didn’t mean to, In4 = Pictures about it popped into my mind, In5 = I found myself acting like I was back at that time, In6= I had waves of strong feelings about it; Av1 = Avoided letting myself get upset when I thought about, Av2 = I stayed away from reminders of it, Av3 = I tried not to think about it, Av4 = Lot of feelings about it, but didn’t deal with them, Av5 = I tried to remove it from my memory, Av6 = I tried not to talk about it; Ha1 = I felt irritable and angry, Ha2 = I was jumpy and easily startled, Ha3 = I had trouble concentrating, Ha4 = Reminders of it caused me to have physical reactions, Ha5 = I felt watchful and on guard; Nb1 = I felt as if it hadn’t happened or it wasn’t real, Nb2 = My feelings about it were kind of numb; Sd1 = I had trouble staying asleep, Sd2 = I had trouble falling asleep, Sd3 = I had dreams about it


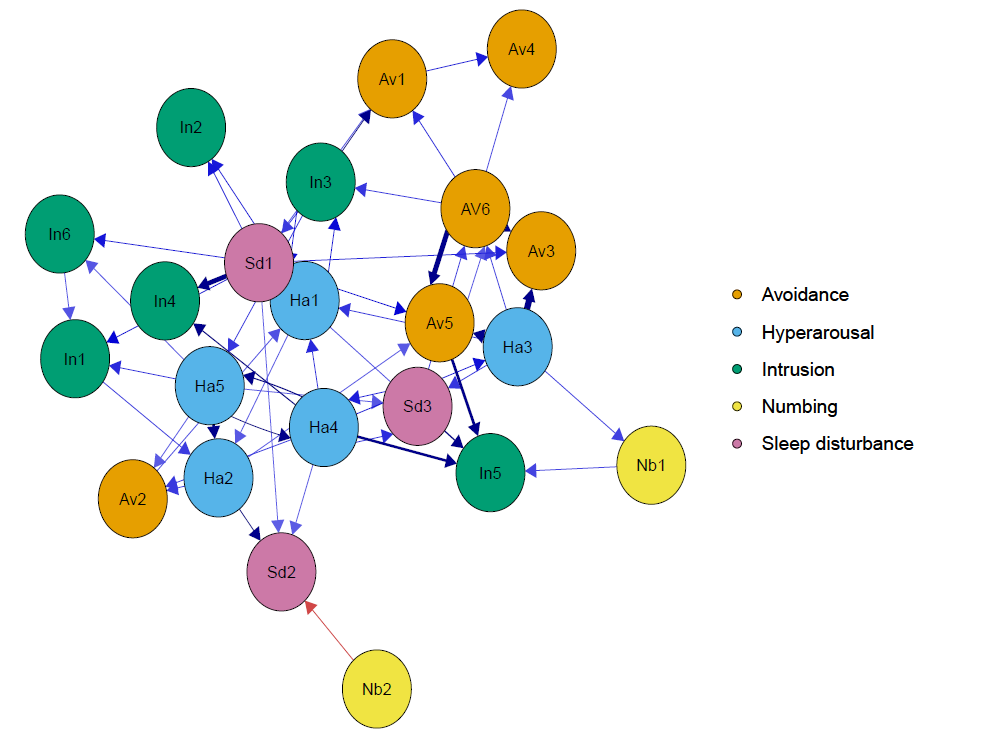


**Supplementary Figure S4***.* The cross-lagged panel network with a single imputed data set (*N* = 737). In1 = Any reminder brought back feelings about it, In2 = Other things kept making me think about it, In3 = I thought about it even when I didn’t mean to, In4 = Pictures about it popped into my mind, In5 = I found myself acting like I was back at that time, In6= I had waves of strong feelings about it; Av1 = Avoided letting myself get upset when I thought about, Av2 = I stayed away from reminders of it, Av3 = I tried not to think about it, Av4 = Lot of feelings about it, but didn’t deal with them, Av5 = I tried to remove it from my memory, Av6 = I tried not to talk about it; Ha1 = I felt irritable and angry, Ha2 = I was jumpy and easily startled, Ha3 = I had trouble concentrating, Ha4 = Reminders of it caused me to have physical reactions, Ha5 = I felt watchful and on guard; Nb1 = I felt as if it hadn’t happened or it wasn’t real, Nb2 = My feelings about it were kind of numb; Sd1 = I had trouble staying asleep, Sd2 = I had trouble falling asleep, Sd3 = I had dreams about it. For visualization, a threshold of β = .05 for the regression weights was chosen.


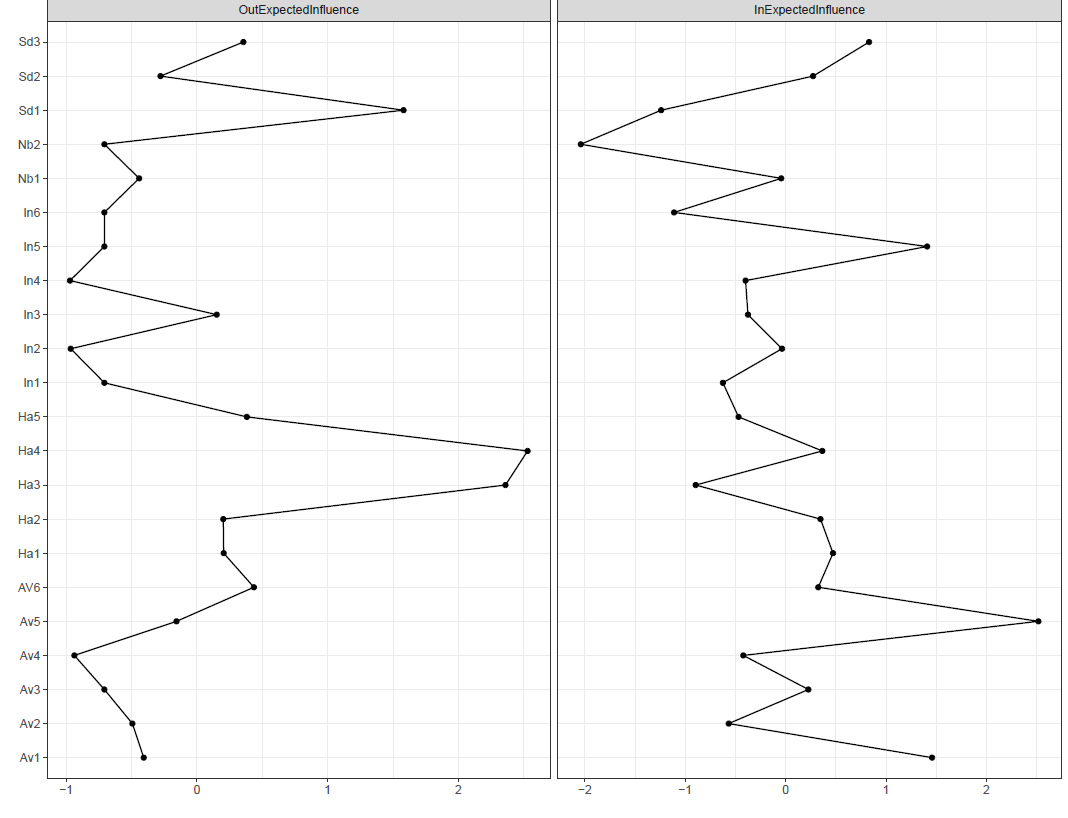


**Supplementary Figure S5**. Centrality estimates (right) using z-values with a single imputed data set (*N* = 737). Greater values indicate greater centrality. In1 = Any reminder brought back feelings about it, In2 = Other things kept making me think about it, In3 = I thought about it even when I didn’t mean to, In4 = Pictures about it popped into my mind, In5 = I found myself acting like I was back at that time, In6= I had waves of strong feelings about it; Av1 = Avoided letting myself get upset when I thought about, Av2 = I stayed away from reminders of it, Av3 = I tried not to think about it, Av4 = Lot of feelings about it, but didn’t deal with them, Av5 = I tried to remove it from my memory, Av6 = I tried not to talk about it; Ha1 = I felt irritable and angry, Ha2 = I was jumpy and easily startled, Ha3 = I had trouble concentrating, Ha4 = Reminders of it caused me to have physical reactions, Ha5 = I felt watchful and on guard; Nb1 = I felt as if it hadn’t happened or it wasn’t real, Nb2 = My feelings about it were kind of numb; Sd1 = I had trouble staying asleep, Sd2 = I had trouble falling asleep, Sd3 = I had dreams about it. For visualization, a threshold of β = .05 for the regression weights was chosen.

**Supplemental Material 2**


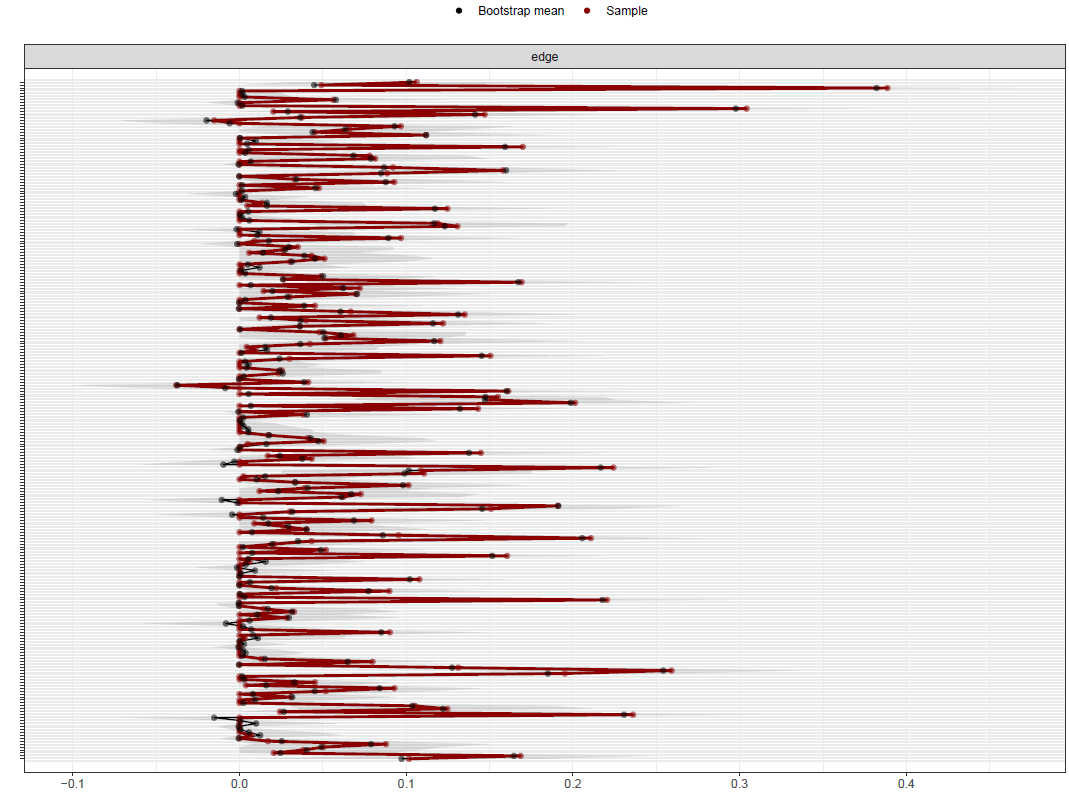


**Supplementary Figure S6.** IES-D interrelation accuracy plots with 1,000 bootstrap iterations for baseline network. Plots show the sample interrelations (i.e., edge weights) with the red dots, the means of the bootstrapped interrelations (i.e., edge weights) with black dots, and the bootstrap confidence intervals.


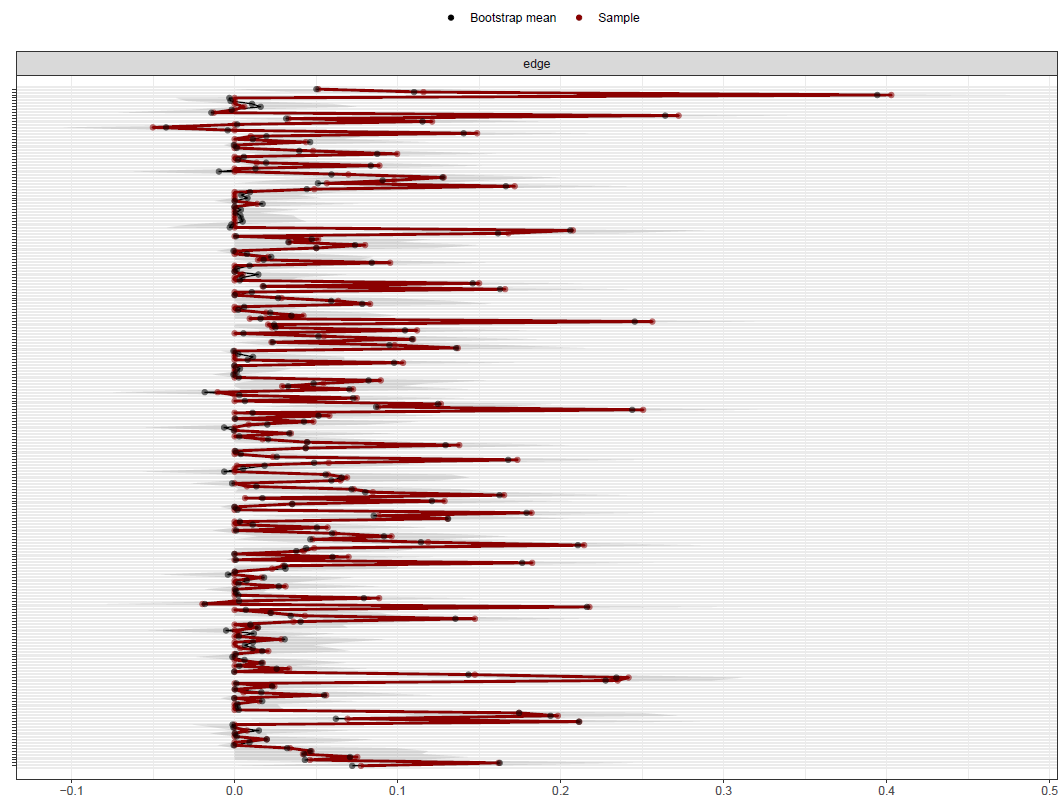


**Supplementary Figure S7.** IES-D interrelation accuracy plots with 1,000 bootstrap iterations for the follow-up network. Plots show the sample interrelations (i.e., edge weights) with the red dots, the means of the bootstrapped interrelations (i.e., edge weights) with black dots, and the bootstrap confidence intervals.


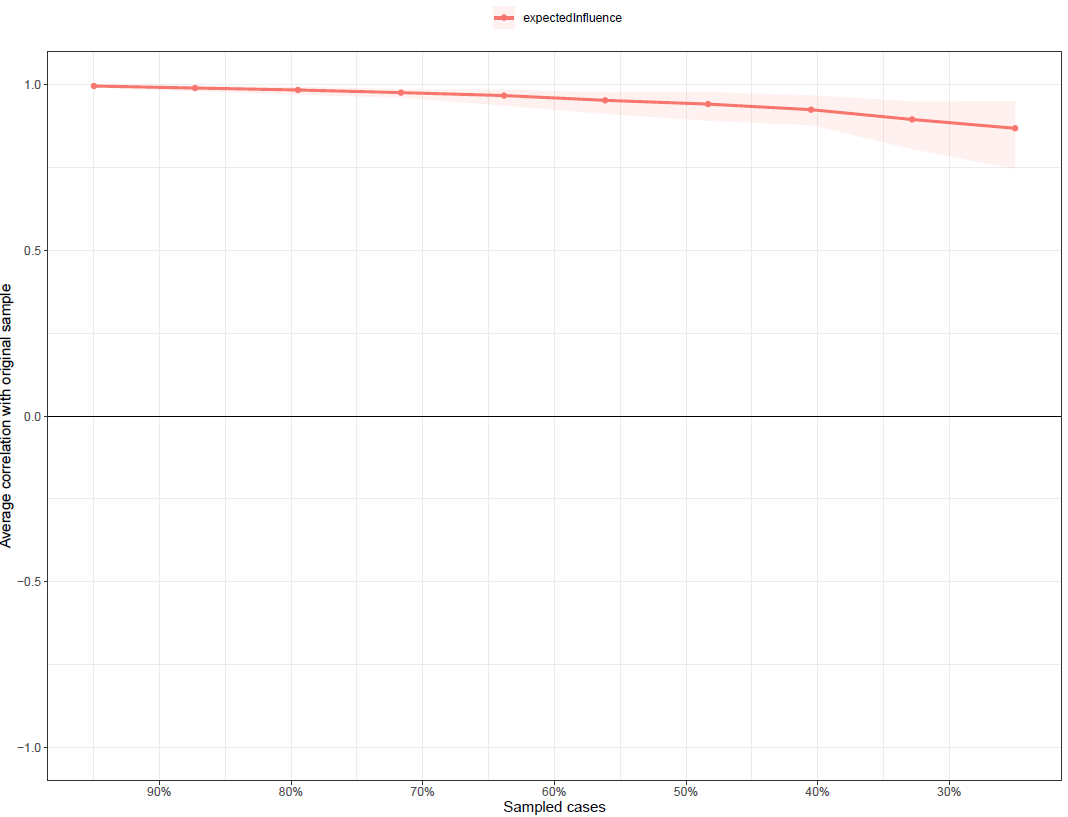
 **Supplementary Figure S8.** Stability of the Expected influence centrality measures for the baseline network.


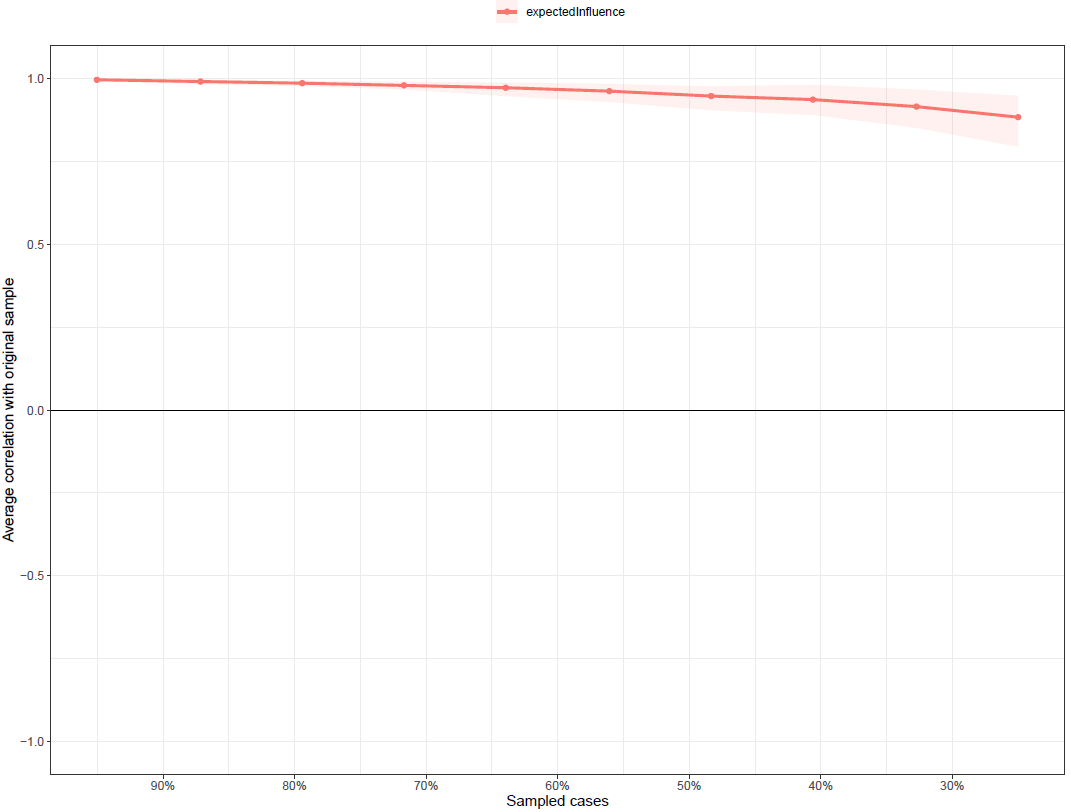
 **Supplementary Figure S9**. Stability of the Expected influence centrality measures for the follow-up network.


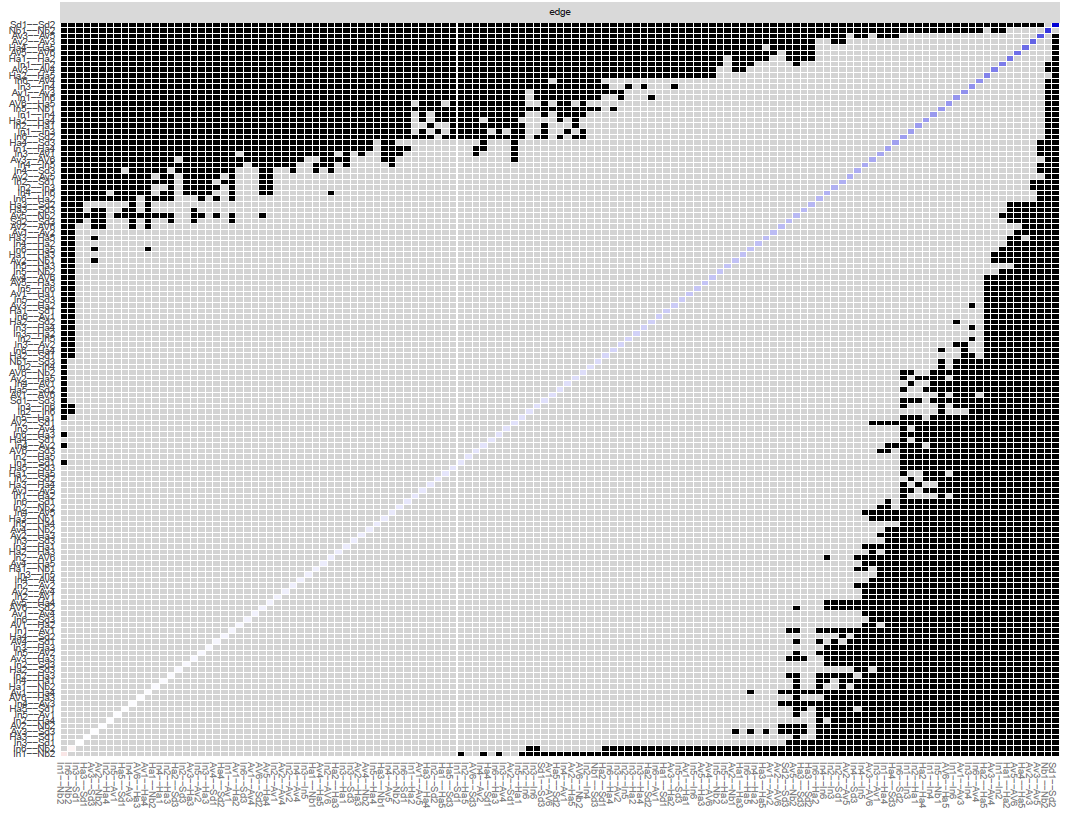
 **Supplementary Figure S10.** Edge weight difference tests for the baseline network. Black boxes indicate edges that significantly differ from each other (*p* < .05). Gray boxes indicate no differences.


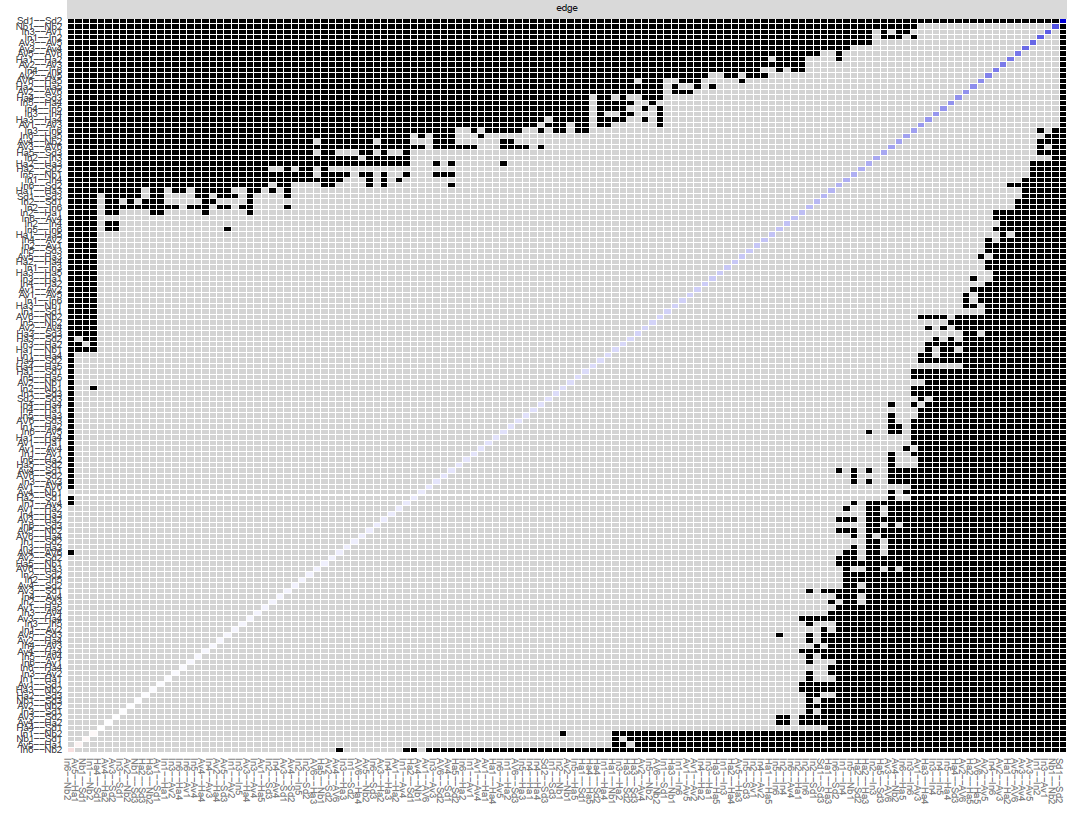
 **Supplementary Figure S11**. Edge weight difference tests for the follow-up network. Black boxes indicate edges that significantly differ from each other (*p* < .05). Gray boxes indicate no differences.


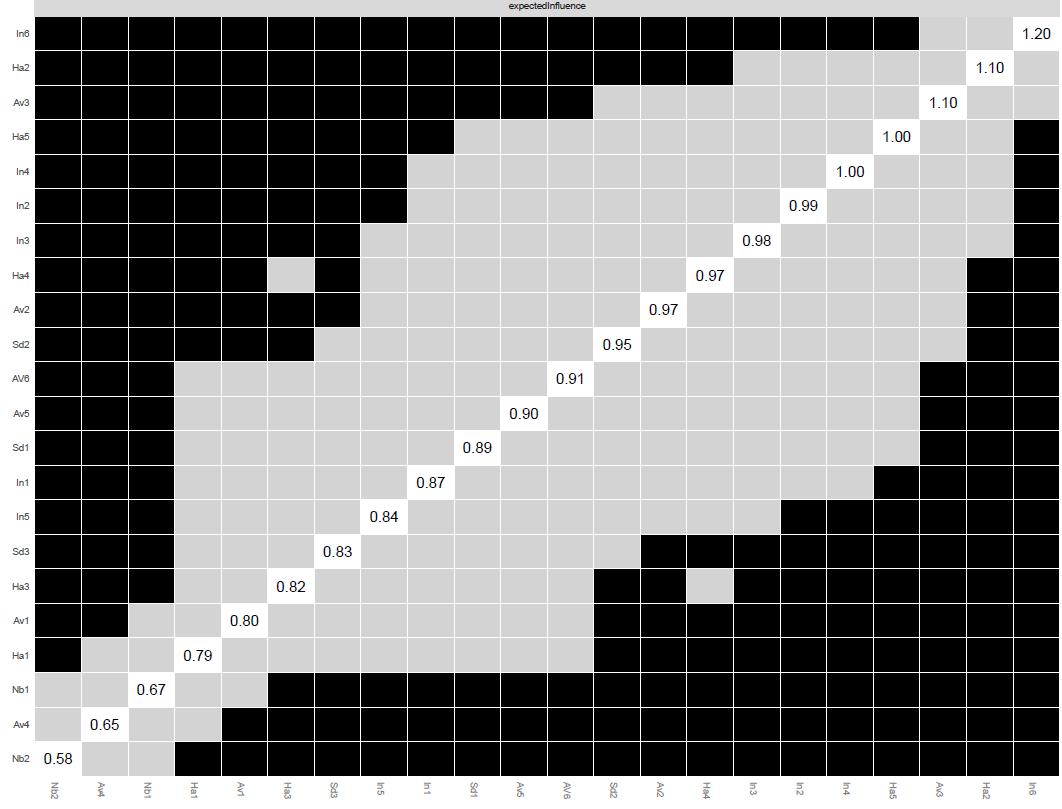
 **Supplementary Figure S12**. Expected influence difference tests for the baseline network. Black boxes indicate edges that significantly differ from each other (*p* < .05). Gray boxes indicate no differences.


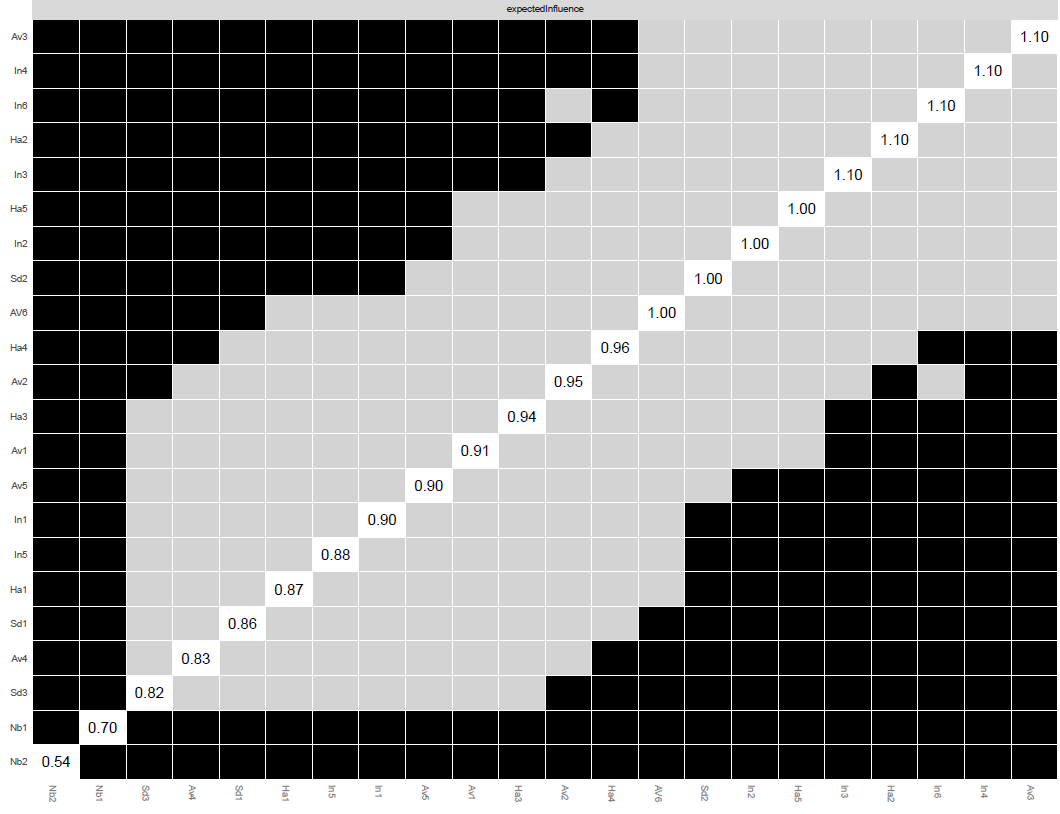
 **Supplementary Figure S13.** Expected influence difference tests for the follow-up network. Black boxes indicate edges that significantly differ from each other (*p* < .05). Gray boxes indicate no differences.


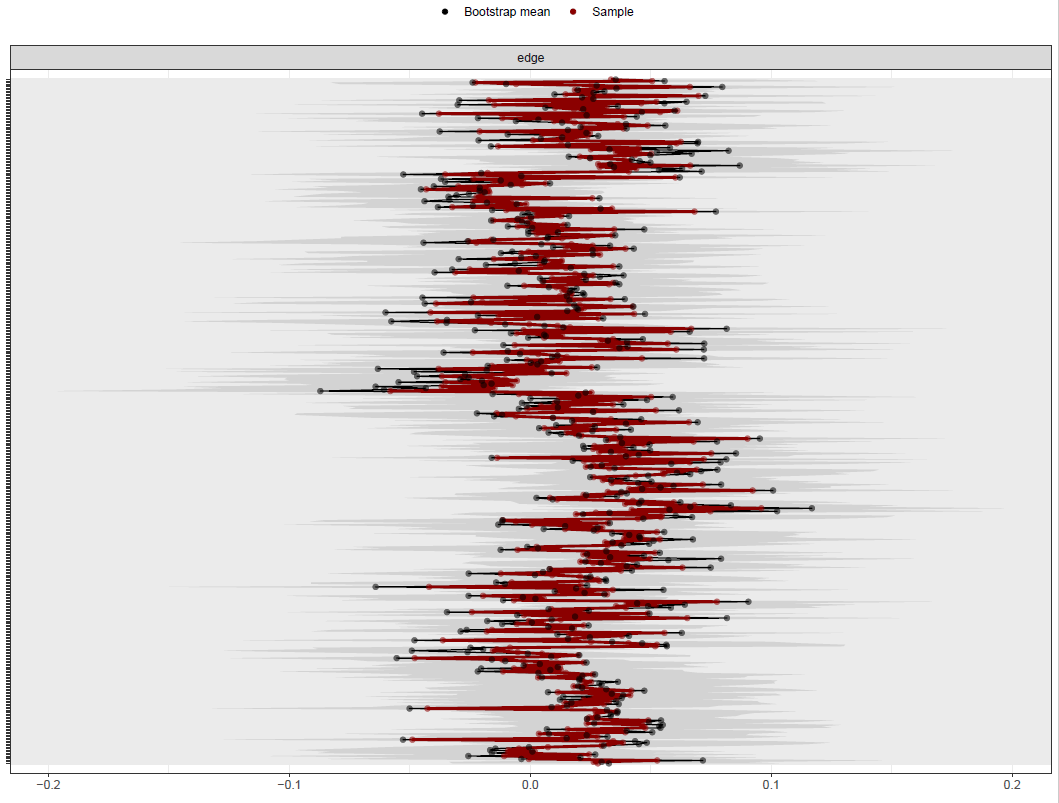


**Supplementary Figure S14.** IES-D interrelation accuracy plots with 1,000 bootstrap iterations for the CLPN. Plots show the sample interrelations (i.e., edge weights) with the red dots, the means of the bootstrapped interrelations (i.e., edge weights) with black dots, and the bootstrap confidence intervals.


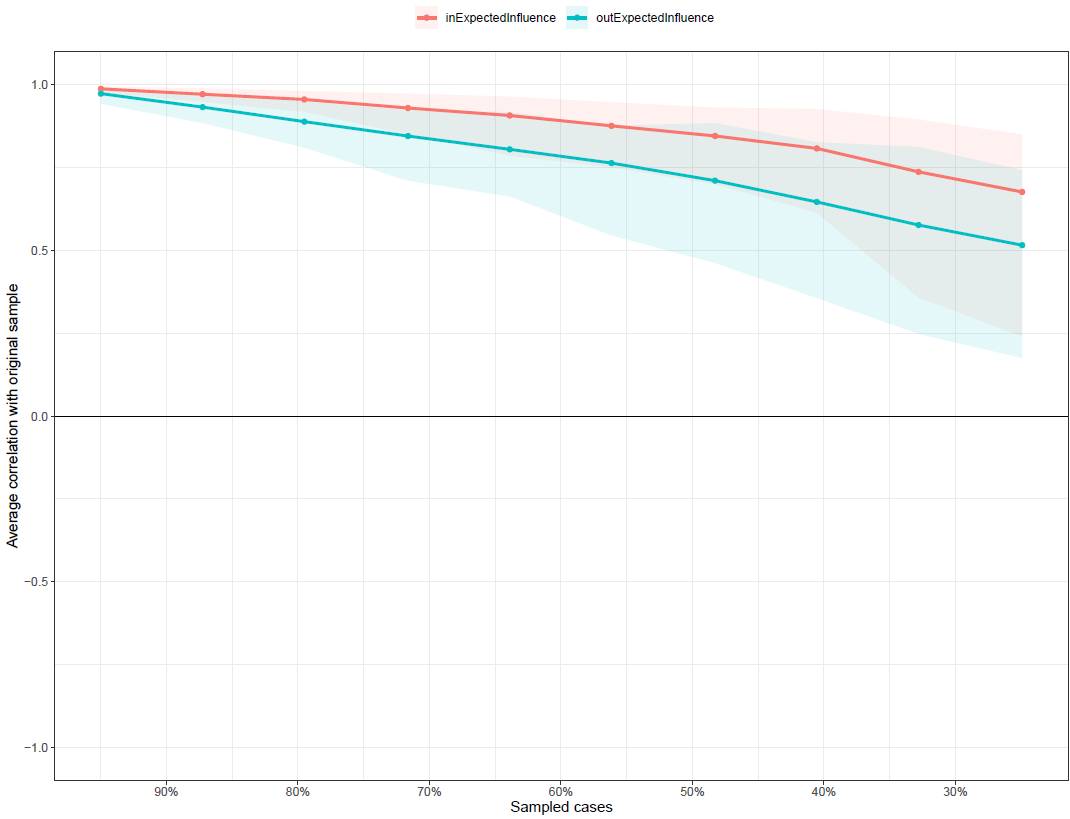
 **Supplementary Figure S15.** Stability of the centrality measures for the CLPN network.


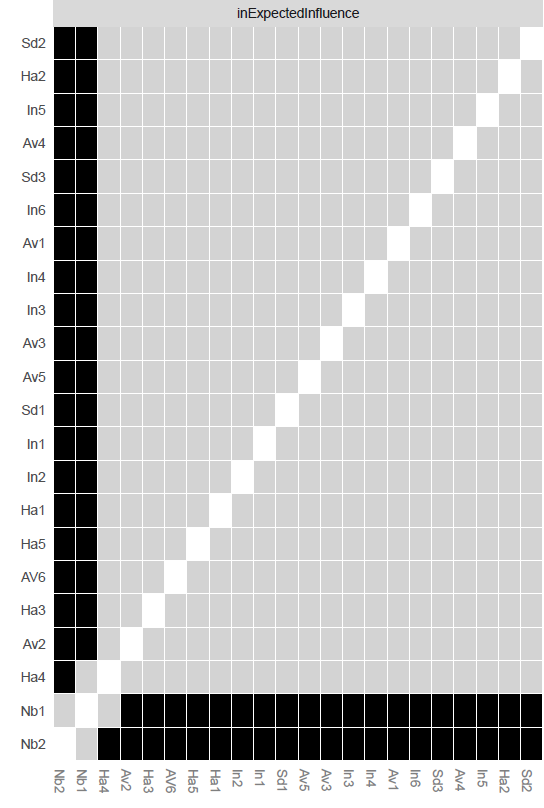


**Supplementary Figure S16**. In-expected-influence difference tests for the CLPN network. Black boxes indicate edges that significantly differ from each other (*p* < .05). Gray boxes indicate no differences.


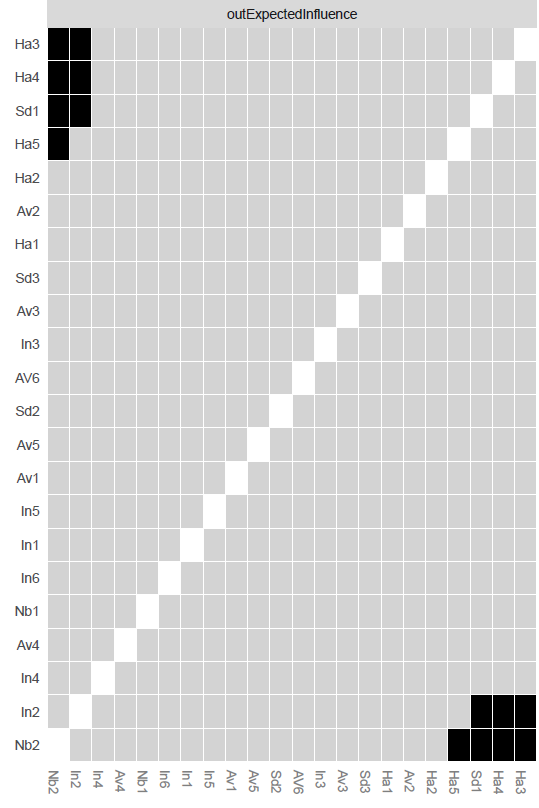


**Supplementary Figure S17.** Out-expected-influence difference tests for the CLPN network. Black boxes indicate edges that significantly differ from each other (*p* < .05). Gray boxes indicate no differences.
